# Liraglutide-Driven Weight Loss Modulates Placental Remodeling in Obese Pregnancies in Mice

**DOI:** 10.3390/cells14242009

**Published:** 2025-12-17

**Authors:** Natassia Rodrigo, Dunja Aksentijevic, Nikayla Patel, Carol A. Pollock, Lana McClements, Sarah J. Glastras

**Affiliations:** 1 Diabetes and Obesity Research Group, Kolling Institute of Medical Research, Sydney, NSW 2065, Australia; carol.pollock@sydney.edu.au (C.A.P.); sarah.glastras@sydney.edu.au (S.J.G.); 2 North Precinct, Sydney Medical School, University of Sydney, Sydney, NSW 2006, Australia; 3 Department of Diabetes, Endocrinology and Metabolism, Royal North Shore Hospital, Reserve Road, St Leonards, Sydney, NSW 2065, Australia; 4 Department of Diabetes and Endocrinology, Nepean Hospital, Sydney, NSW 2747, Australia; 5 William Harvey Research Institute, Barts and the London Faculty of Medicine and Dentistry, Queen Mary University of London, London EC1M 6BQ, UK; d.aksentijevic@qmul.ac.uk (D.A.); n.p.patel@qmul.ac.uk (N.P.); 6 School of Life Sciences, Faculty of Science, University of Technology Sydney, Sydney, NSW 2007, Australia; lana.mcclements@uts.edu.au

**Keywords:** reproductive health, placenta, pre-conception, liraglutide, diet

## Abstract

**Highlights:**

**What are the main findings?**

- Maternal obesity caused by a high-fat diet alters the placental physiological profile through oxidative stress (increased *Nox4*), inflammation (*Il6*), and dysregulated metabolism.
- Pharmacological treatment with liraglutide prior to pregnancy led to normalization of gene expression of metabolic regulators (*PGC1alph*a, *FAS*) and metabolic intermediates (glutamine, acetate, alanine, lactate) but not oxidative stress nor inflammation in the placenta.
- Pre-conception dietary change exerted a similar effect to liraglutide treatment; however, it led to a reduction in placental oxidative stress (*NOX4*) and inflammation (*IL6*) and was not associated with excessive gestational weight gain.
- Diet modulation after pregnancy induced signals of placental stress, including low glutamine and carnitine levels, which can be associated with growth restriction and reduced fatty acid metabolism.

**What are the implications of the main findings?**

- Weight modulation prior to pregnancy, either by diet changes or through GLP-1 receptor agonist therapy leads to altered metabolic and inflammatory profiles in the placenta.

**Abstract:**

Background: The placenta stands at the maternal–fetal interface and is a key organ regulating the intrauterine environment. In pregnancies exposed to obesity, placental function, signaling, and nutrient handling are adversely altered. Pre-conception weight loss is a potential intervention to alter an obesogenic milieu of pregnancy, which we investigated in a mouse model of maternal obesity using diet or administration of the glucagon-like peptide-1 (GLP-1) receptor agonist liraglutide. Methods: Pre-pregnancy weight loss in *C57BL/6* high-fat diet (HFD)-fed dams was induced in the pre-pregnancy period by switching diet from HFD to chow diet or administering liraglutide (0.3 mg/kg/day subcutaneously for 4 weeks) whilst continuing HFD. In addition, a group of HFD-fed dams were switched to chow diet post-conception. The metabolomic profile and gene expression within the placenta was compared at day 18–20 of gestation. Results: ^1^H NMR spectroscopy metabolomic analysis of placenta of HFD mice showed an altered amino acid metabolomic profile, with lower aspartate, glutamate, and glutamine levels compared to the placenta of chow-fed mice (*p* < 0.05). Meanwhile, gene expression analysis identified both oxidative stress and inflammation in the placentas of HFD-fed dams. Whilst dietary modification alone was sufficient to reduce markers of oxidative stress and inflammation, liraglutide treatment modulated pathological changes, including placental metabolic stress but not inflammation. Conclusions: These findings highlight the importance of dietary or pharmacological interventions in the pre- or immediate post-conception period, with pre-conception offering a critical window to reduce aberrant placental changes induced by obesity.

## 1. Introduction

The global epidemic of obesity is reaching alarming proportions, with a tripling of incidence over the past four decades [1]. This has led to a concomitant increase in the complications of obesity, which are manifold and ubiquitous, with increased incidence of cardiovascular disease, diabetes mellitus, musculoskeletal disorders, respiratory disorders, and liver and chronic kidney disease [2]. Of particular concern, the prevalence of obesity is increasing in women of reproductive age, conservatively estimated to affect 21% of women in the world by 2025 [3]. Women of reproductive age are a particularly vulnerable group as the effects of obesity are manifested in pregnancy, with known increased risks of short-term and long-term complications for both the mother and the offspring [4,5].

An accumulating body of evidence increasingly implicates maternal obesity and gestational weight gain as a risk factor for childhood obesity and overweight, with a 4-fold increased risk demonstrated by meta-analysis [6]. A UK study demonstrated that all-cause mortality was 1.35 times higher in offspring of obese mothers compared to offspring of mothers with a normal BMI, with a 1.3 times greater risk of cardiovascular disease [7]. A large population study demonstrated a 3.5 times higher hazard of developing diabetes mellitus in the offspring of mothers with obesity [8]. This risk appears proportional to the degree of maternal obesity, with a previous study demonstrating that the risk of hypertension in offspring increased by 5% for every increment in maternal BMI of 1 unit [9]. Mechanistically, maternal obesity compounds physiological pregnancy changes in maternal metabolism to increase inflammation and blood lipids, impacting fetal development [10].

These issues were explored in our previous work. We utilized a mouse model of maternal obesity and insulin resistance to explore the impact of weight modulation on fertility and maternal pregnancy outcomes [11]. We demonstrated that liraglutide therapy in the pre-conception period was an effective method to induce weight reduction and improve glucose tolerance, despite ongoing HFD, in the pre-conception period, with a resultant improvement in fertility. Following its cessation, despite catch-up weight gain, liraglutide was effective in reducing glucose intolerance in late gestation. We showed that a pre-conception diet switch from an HFD to a chow diet was also effective in achieving weight reduction and improved glucose tolerance, persistent during pregnancy. To date, the impact of pre-conception maternal weight modulation on the effects on placental health has not been explored.

The placenta is a crucial regulating organ that is formed in early pregnancy and is situated at the interface between the maternal and fetal circulation, yet its role in developmental programming of the fetus has not been fully explored [12]. Maternal obesity is associated with increased inflammatory infiltration of the placenta, coupled with increased expression of oxidative stress markers, which cumulatively impact placental nutrient transport and placental function [13]. Further, mitochondrial dysfunction in placentas exposed to obesity leads to reduced energy production [13]. These changes culminate in reduced nutrient and oxygen supply to the fetus, leading to growth restriction [13]. We postulate that maternal body weight reduction prior to pregnancy in obese mothers may facilitate improved maternal, placental, and offspring health.

The aim of this study was to determine if weight loss prior to pregnancy, with diet modification or liraglutide, versus diet change in pregnancy alters placental metabolomic and transcriptomic profiles in a mouse model of obesity. We hypothesized that placental inflammation, oxidative stress, and metabolism would be improved in the face of a reduced burden of maternal obesity. Given the recent evidence demonstrating the utility of pre-pregnancy weight modulation on fertility and metabolic health in late pregnancy, we expected that pre-pregnancy weight modulation would beneficially alter the placental metabolomic profile and gene expression of selected redox and inflammatory markers compared to weight intervention during pregnancy.

## 2. Methods

### 2.1. Animals

This study utilized a mouse model of maternal obesity, which we have previously described [11,14]. Briefly, female *C57Bl/6* mice were obtained at 4 weeks of age (Kearns Facility, Kolling Institute, Royal North Shore Hospital, St Leonards, Sydney, NSW, Australia, N = 96). They were housed in the Kearns Animal Facility of Kolling Institute with a stable environment maintained at 22 ± 1 °C with a 12 h light–dark cycle and humidity between 40–60% in groups of 3–4 mice per cage to allow for socialization, with ad libitum access to food and water. Animal monitoring, including weighing, occurred on a weekly basis. Procedures were conducted according to approved standard operating procedures (SOP’s) by the Animal Care and Ethics Committee (AEC) of the Northern Sydney Local Health District (RESP/18/148). The study was conducted in compliance with the Australian Code of Practice for the Care and Use of Animals for Scientific Purposes as well as the Animal Research: Reporting of In Vivo Experiments (ARRIVE) guidelines [15].

**Figure 1:**
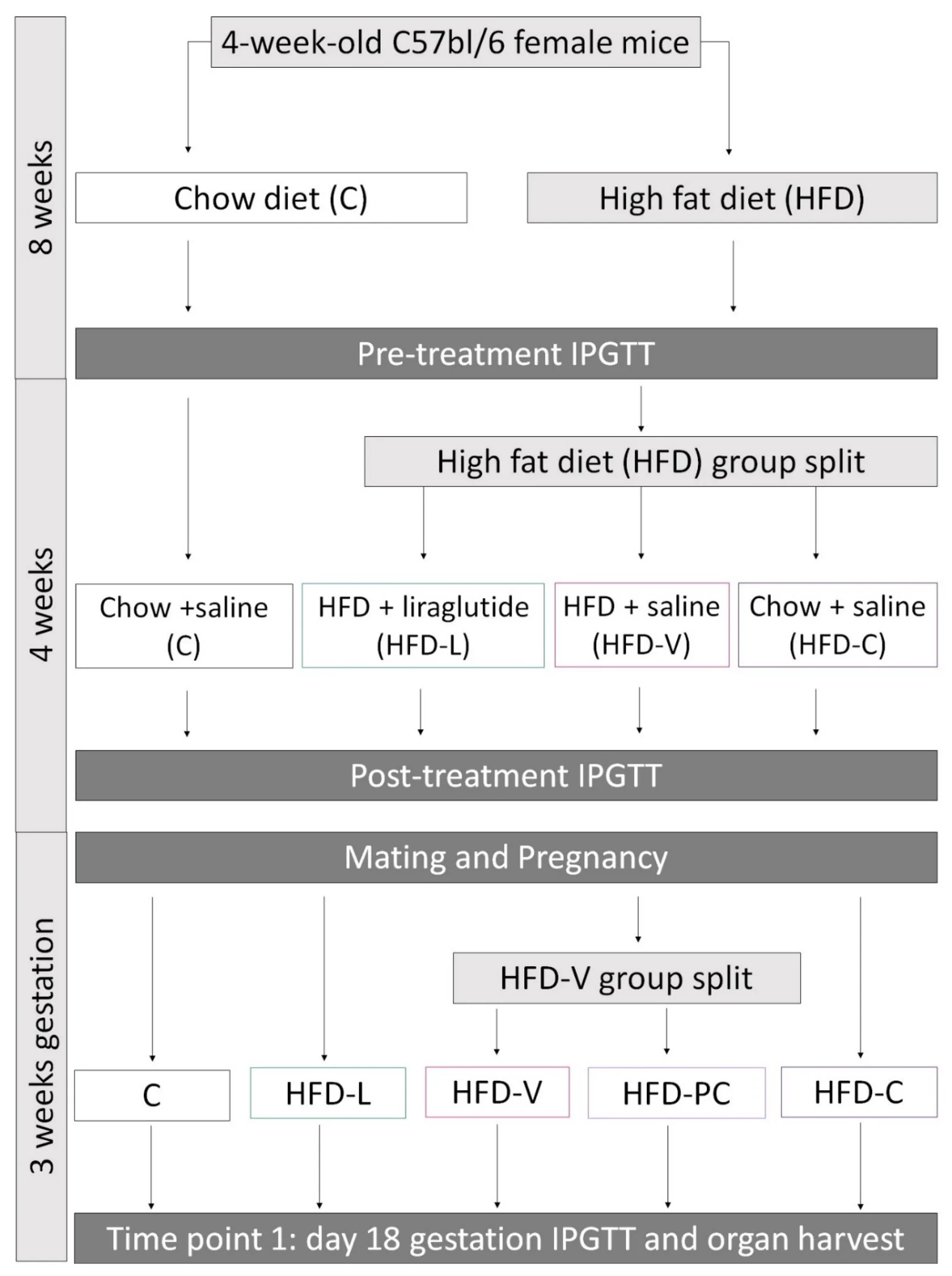
Study design charting the progression through the pre-conception and pregnancy periods. C: control/chow-fed mice. HFD: high-fat-diet-fed mice. IPGTT: intraperitoneal glucose tolerance test. HFD-L: high-fat-diet-fed mice plus liraglutide therapy. HFD-V: high-fat-diet-fed mice plus saline. HFD-C: high-fat-diet-fed mice changed to chow. HFD-PC: high-fat-diet-fed mice changed to chow diet after pregnancy confirmed.

Female mice were randomly allocated 4:1 (to take into account the 5 mouse groups required, 4 of which were HFD-fed) into 2 groups to receive either a high-fat diet (HFD comprising 20 KJ/g, 43% fat, and 21% protein; SF04-001; Specialty Feeds, Glen Forrest, WA, Australia), to induce obesity or a chow diet (11 KJ/g, 14% fat, 21% protein) for 8 weeks (control) (Figure 1). Sample size was determined based on fertility and fecundity outcomes in our previous work [11]. Baseline intraperitoneal glucose tolerance tests were performed to establish a baseline, the methodology of which has been previously described [11]. Researchers were blinded to the treatment group at the time of IPGTT. Briefly, mice were weighed and fasted for 6 h. A treatment of 50% glucose (2 g/Kg) was administered via intraperitoneal injection, and blood glucose levels were measured via tail tip sampling at 0 min, 15 min, 30 min, 60 min, 90 min, and 120 min using a Roche AccuCheck Performa Meter, Roche, Basel, Switzerland.

Thereafter, the HFD group was divided equally into 4 groups:

(1) HFD-V: HFD–vehicle (saline solution);
(2) HFD-L: HFD in combination with liraglutide by daily subcutaneous injections;
(3) HFD-C: HFD with switch to chow in the pre-conception period;
(4) HFD-PC: HFD with switch to chow once pregnancy was confirmed.

As previously described, liraglutide was administered to the HFD-L group via a dose incrementation of 3 days of 0.1 mg/kg/day, followed by 3 days of 0.2 mg/kg/day, to a full treatment dose of 0.3 mg/kg/day to reduce the adverse effects of liraglutide such as nausea and gastrointestinal discomfort [16]. The four remaining groups received weight-based normal saline injections, (i.e., the vehicle solution). All mice in a single cage underwent the same grouping and subsequent treatment procedure. Stratified randomization using body weight was used to reduce allocation bias. IPGTT was repeated after 4 weeks of treatment with either liraglutide or saline injections.

Following a one week wash-out period, to minimize the risk of teratogenic effects of liraglutide, dams underwent male co-housing (male to female ratio 1:3) for 3 days. The presence of a vaginal plug and daily incremental weight gain indicated pregnancy. If pregnancy was not achieved, 2 further cycles of mating were attempted. A priori, mice that did not fall pregnant were excluded. Once pregnancy was confirmed, pregnant female mice were housed individually until the end of gestation [11]. Further, upon verification of pregnancy, the HFD-PC group switched to a chow diet. HFD-L and HFD-V mice were continued on an HFD. HFD-C and control mice were continued on a chow diet. At day 18 of gestation, pregnant mice underwent an IPGTT to assess glucose tolerance in late gestation. Following a 4 h fast, 8 pregnant dams were sacrificed at gestational day 19–20. Euthanasia procedure involved anaesthesia with isoflurane, with cardiac puncture to obtain blood samples. Blood was immediately centrifuged and serum was separated. Placenta were harvested, weighed, and frozen in liquid nitrogen. Serum and placental samples were stored at −80 degrees Celsius. The number of fetuses present were counted. The remaining pregnant dams were allowed to deliver their litters. Maternal age at delivery, litter sizes at birth, and the number of surviving offspring by day 3 of life were recorded. At the time of euthanasia, mice were allocated a study code to blind researchers to the treatment group for all further analyses, including tissue and bioassay analyses.

### 2.2. Real Time PCR (RT-PCR)

RNA was extracted from placental tissue using an RNeasy Plus Mini Kit (Qiagen, Redwood City, CA, USA), and purified total RNA was used as a template to generate cDNA using an iScript cDNA Synthesis Kit (Biorad, Hercules, CA, USA). RT-PCR was performed using a QuantiNova PCR kit (Qiagen, Hilden, Germany). RT-PCR was carried out with a QuantStudio 12K Flex Real-Time PCR System (Thermo Fisher Scientific, Waltham, MA, USA). The cycle threshold (Ct) value was analyzed using the delta–delta–Ct method. Results were normalized to 18S and expressed as fold change. RT-PCR primer sequences used are listed in Table 1. Obesity-related metabolic markers included fatty acid synthase (FAS), peroxisome-proliferator-activated receptor alpha (PPAR-α), and peroxisome-proliferator-activated receptor gamma coactivator 1-alpha (PGC1α) [17,18,19]. Oxidative stress was assessed by the mRNA expression of NADPH oxidase 4 (NOX-4) and inflammation assessed by interleukin-6 (IL-6) [20,21].

### 2.3. ^1^H Nuclear Magnetic Resonance Spectroscopy Metabolic Profiling

Placentas were analyzed using a previously described methodology [22,23]. Briefly, frozen and pulverized placentas were exposed to methanol/water/chloroform dual-phase extraction. The aqueous upper phase was separated from the chloroform and protein fractions. A total of 30 mg chelex-100 was added to chelate paramagnetic ions, vortexed, and centrifuged at 3600 RPM for 5 min at 4 °C. The supernatant was added to a fresh Falcon tube containing 10 µL of universal pH indicator solution followed by vortexing and lyophilization. Dual-phase-extracted metabolites were reconstituted in 600 µL of deuterium oxide [containing 8 g/L NaCl, 0.2 g/L KCl, 1.15 g/L Na_2_HPO_4_, 0.2 g/L KH_2_PO_4_, and 0.0075% *w*/*v* trimethylsilyl propanoic acid (TSP)] and adjusted to pH ≈ 6.5.

A vertical-bore, ultra-shielded Bruker 14.1.T (600 MHz) spectrometer, Bruker, Billerica, MA, USA, with a BBO probe at 303 K was used for sample analysis. Spectra were acquired with the Bruker noesygppr1d pulse sequence with 128 scans, 4 dummy scans, and a 20 ppm sweep width, an acquisition time of 2.6 s, a pre-scan delay of 4 s, a 90° flip angle, and an experiment duration of 14.4 min. The TopSpin (version 4.0.5) software was used for data acquisition and metabolite quantification. FIDs were multiplied by a line broadening factor of 0.3 Hz and Fourier-transformed. Phase and automatic baseline correction were applied. Chemical shifts were normalized by setting the TSP signal to 0 ppm. In terms of glycogen quantification, given that it is a large macromolecule containing multiple glucose monomers, we measured several mobile ^1^H in the glucose monomers that are present in the nuclear magnetic resonance spectroscopy (NMR) peak, normalized to the reference. Peaks of interest were integrated automatically using a pre-written integration region text file and then manually adjusted where required. Assignment of metabolites to their respective peaks was carried out based on previously obtained in-house data, confirmed by chemical shift, and using Chenomx NMR Profiler Version 8.1 (Chenomx, Edmonton, AB, Canada). Peak areas were normalized to the TSP peaks, and metabolite concentrations were quantified per gram of tissue wet weight.

### 2.4. Statistical Methods

All results were expressed as the mean ± standard error of the mean (SEM). Normality of data distribution was examined using Shapiro–Wilk’s and the Kolmogorov–Smirnov tests. Differences in means were compared using one-way analysis of variance (ANOVA), with Tukey’s post hoc tests, including placental metabolomic data [18]. Area under the curve (AUC) was determined using the trapezoidal rule for IPGTT results [24]. Outliers were excluded using the Rout method. All analyses were carried out using GraphPad Prism 9.0 (GraphPad Software, San Diego, CA, USA), and *p* value < 0.05 was considered statistically significant.

## 3. Results

### 3.1. Efficacy of the Mouse Model of Maternal Obesity

As we have previously reported, following the 8-week period of HFD feeding, there was a significant difference in maternal weight between chow- and HFD-fed mice (chow-fed mice 21.3 ± 0.5 g vs. HFD-fed mice 23.8 ± 0.3 g, *p* < 0.01) [11]. This weight difference translated to differences in glucose tolerance on IPGTT, with a greater area under the curve (AUC) in the HFD-fed mice compared to the chow-fed mice (*p* < 0.0005) [11].

### 3.2. Pre-Conception Maternal Weight Change and Metabolic Outcomes

Following liraglutide injections, significant weight loss was achieved in the HFD-L group compared to the HFD-V group, such that the body weight post-liraglutide treatment and pre-conception was similar to that of the control group (HFD-L 24.8 ± 0.8 g vs. HFD-V 27.4 ± 1.1 g, *p* < 0.05, Table 2). Following diet change in the pre-conception period, the HFD-C group also had a significant weight loss compared to HFD-V mice (HFD-C 23.3 ± 0.5 g vs. HFD-V 27.4 ± 1.1 g, *p* < 0.001, Table 2), resulting in a similar body weight to the control and HFD-L groups by the end of the 4-week period.

### 3.3. Late-Gestation Maternal Weight Change and Metabolic Outcomes

At late gestation (gestational day 18–20), the body weight changes seen in the control, HFD-L, HFD-V, and HFD-C groups have previously been reported [11] and are recapitulated here for comparison, with the previously unreported anthropometric measures for the HFD-PC group now included (Table 2) [11]. Body weight in late gestation was significantly higher in the HFD-L group (*p* < 0.05) and HFD-V group (*p* < 0.0005) compared to the HFD-PC group. HFD-PC mice had the lowest gestational weight gain, with 3.2 ± 0.4 g gained by late gestation, significantly less than all other groups (control: 5.7 ± 0.5 g, HFD-V: 5.3 ± 0.7 g, HFD-L: 6.8 ± 0.6 g, HFD-C: 5.0 ± 0.4 g; *p* < 0.05, *p* < 0.0001 *p* < 0.01, *p* < 0.05, respectively).

In late gestation, the HFD-PC group had reduced glucose tolerance compared to the control group and the HFD-C group (AUC HFC-PC 98.3 ± 7.0 vs. control: 81.7 ± 4.5 mmol/L/min or HFD-C: 83.8 ± 7.2 mmol/L/min, both *p* < 0.05, Table 2). In addition, glucose tolerance was impaired in the HFD-L and HFD-V groups (HFD-L: 109.5 ± 11.3 mmol/L/min and HFD-V 122.3 ± 7.1 mmol/L/min *p* < 0.0001, *p* < 0.05, respectively) compared to the control, as previously described [11]. There was a significant difference in insulin levels between HFD-PC and all other groups (Table 2). HOMA-IR was significantly higher in the HFD-PC group compared to the control group (*p* < 0.005). With respect to lipids, levels of non-esterified fatty acids (NEFAs), total cholesterol, HDL, and LDL were similar between the HFD-PC and control groups, except for lower serum triacylglyceride (TAG) in the HFD-PC group (*p* < 0.005 vs. control, Table 2).

There were no differences in the fetal weight (average of all groups, 0.46 ± 0.04 g). The number of fetuses present at late gestation was significantly lower in the HFD-PC group compared to the control group (HFD-PC: 7.4 ± 0.4 vs. control: 9.9 ± 0.7, *p* < 0.05) and was similar to the HFD-V (7.3 ± 0.4) and HFD-L (7.2 ± 0.4) groups.

Mean maternal age at placental collection was 22.68 ± 0.26 weeks, similar across all groups. Across all treatments, and in comparison to the control, placental weights were mostly comparable between the groups, with the exception of the placentas of HFD-C, which were bigger than the control or the other groups (0.11 ± 0.018 g vs. 0.13 ± 0.021 g, *p* < 0.005, Table 2).

At the time of birth, there was a significant difference in the live birth litter sizes between the HFD-PC and the control groups (HFD-PC: 5.2 ± 0.4 vs. control: 6.6 ± 0.4, *p* < 0.05); HFD-V has the lowest live birth litter size (4.7 ± 0.4). The number of offspring surviving after birth was significantly lower in the HFD-PC group compared to the controls (3.7 ± 0.5 vs. 5.8 ± 0.7, *p* < 0.05), with a similar infanticide rate in the HFD-V, HFD-L, and HFD-PC groups compared to the control or HFD-C groups (*p* < 0.05).

### 3.4. Placental mRNA Expression of Metabolic, Oxidative Stress, and Inflammatory Markers

**Figure 2:**
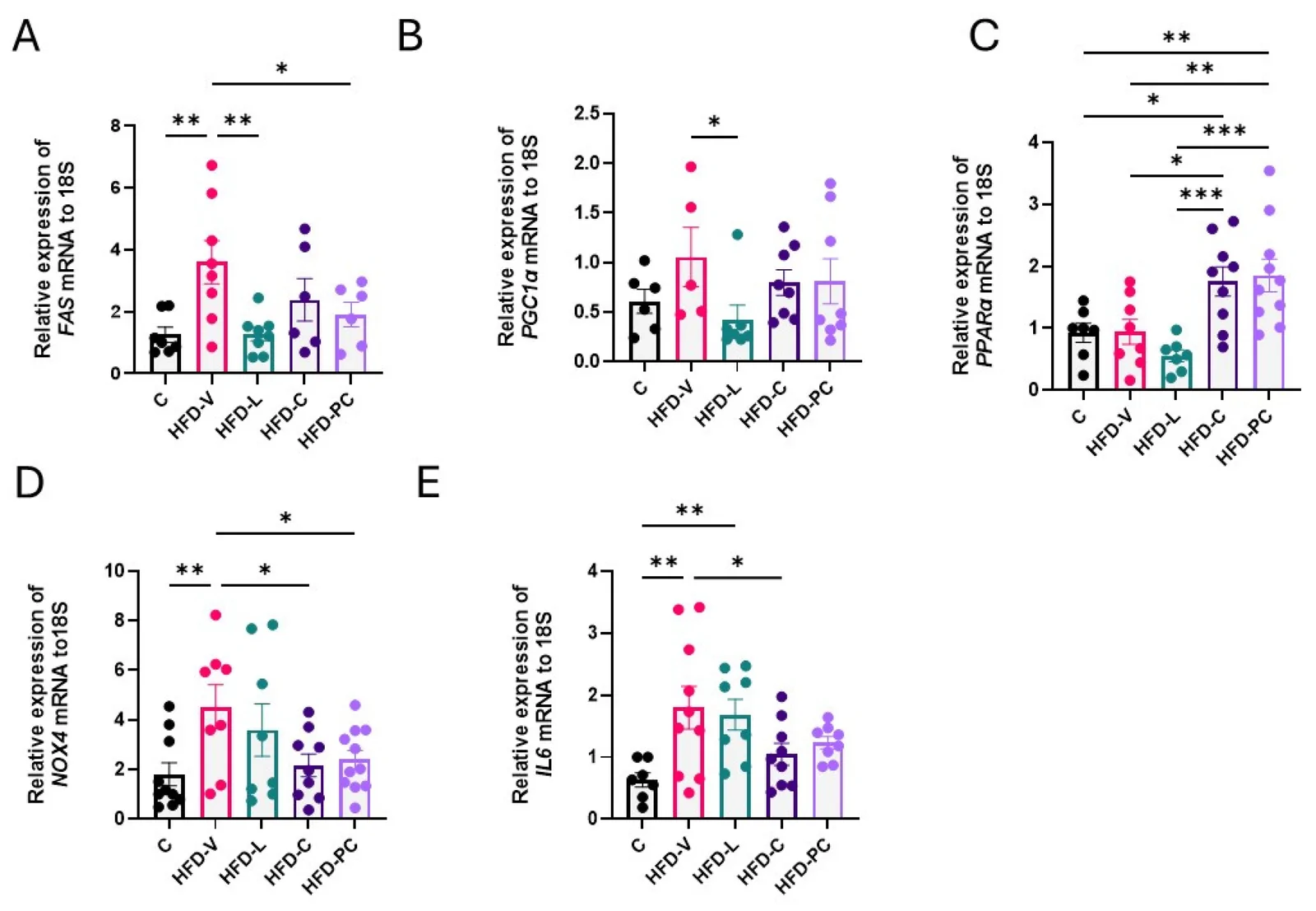
RT-PCR metabolic, oxidative stress, and inflammatory markers in placental tissue, expressed relative to 18S. (**A**) *FAS* mRNA expression. (**B**) *PGC1α* mRNA expression. (**C**) *PPARα* mRNA expression. (**D**) *NOX4* mRNA expression. (**E**) *IL-6* mRNA expression. N = 7–10 per group, ANOVA results expressed as mean ± SEM, * *p* < 0.05, ** *p* < 0.005 *** *p* < 0.0005. C: chow, HFD-V: HFD-vehicle, HFD-L: HFD in combination with liraglutide by daily subcutaneous injections, HFD-C: HFD switched to chow in the pre-conception period, HFD-PC: HFD switched to chow once pregnancy was confirmed.

Dams consuming a high-fat diet (HFD-V) had higher placental mRNA expression of fatty acid synthase (*FAS*) compared to the control (*p* < 0.005, Figure 2A). Pre-conception liraglutide treatment (HFD-L) and post-conception diet switch to chow (HFD-PC) facilitated normalization of *FAS* expression to similar levels as the control group (HFD-L, HFD-C, HFD-PC similar to control, *p* = N.S.; Figure 2A).

HFD alone did not increase *PGC1α* mRNA expression (HFD-V) vs. the control group, although there was a trend towards an increase (*p* = 0.14; Figure 2B). However, pharmacological intervention significantly lowered mRNA expression of *PGC1α* in the HFD-L group compared to the HFD-V group (*p* < 0.05; Figure 2B). There was no difference in *PPAR-α* mRNA expression between the HFD-V group and the control group (Figure 2C). However, the HFD-C and HFD-PC groups had higher *PPAR-α* mRNA expression compared to the control, HFD-V, or HFD-L groups (*p* < 0.05, *p* < 0.05, *p* < 0.0005, respectively). *NOX-4* had higher mRNA expression in the HFD-V group compared to the control (*p* < 0.005, Figure 2D). Diet modulation pre- or post-conception normalized this increase (*p* < 0.05). Liraglutide therapy did not lower *NOX-4* mRNA expression, with no difference between the HFD-L and HFD-V groups (*p* > 0.05). *IL-6* mRNA expression was significantly increased in the HFD-V and HFD-L groups compared to the controls (both *p* < 0.005, Figure 2B), whereas diet modulation pre-conception led to a reduction in *IL-6* mRNA expression (HFD-V vs. HFD-C, *p* < 0.05, Figure 2E).

### 3.5. ^1^H Nuclear Magnetic Resonance Spectroscopy Metabolic Profiling of Placenta

**Figure 3:**
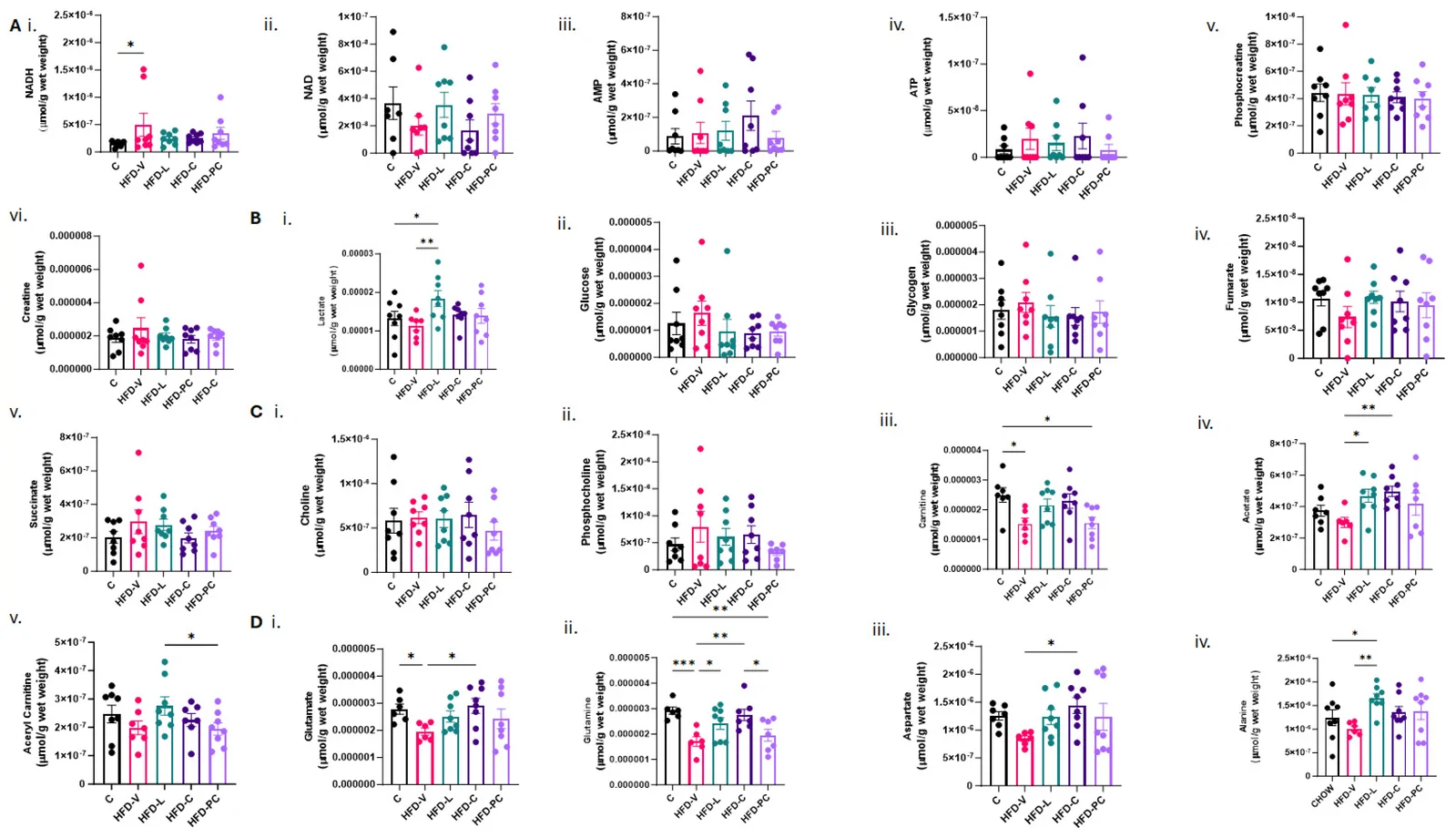
Placental metabolomic profile. (**A**) Redox and energetics. (**i**) NADH. (**ii**) NAD. (**iii**) AMP. (**iv**) ATP. (**v**) Phosphocreatine. (**vi**) Creatine. (**B**) Carbohydrate metabolism and TCA Cycle. (**i**) Carbohydrate metabolism: lactate. (**ii**) Carbohydrate metabolism: glucose. (**iii**) Carbohydrate metabolism: glycogen. (**iv**) TCA cycle: fumarate. (**v**) TCA cycle: succinate. (**C**) Lipid metabolism. (**i**) choline. (**ii**) Phosphocholine. (**iii**) Carnitine. (**iv**) Acetate. (**v**) Aceryl carnitine. (**D**) Amino acid metabolism. (**i**) Glutamate. (**ii**) Glutamine. (**iii**) Aspartate. (**iv**) Alanine. N = 6–8 per group, ANOVA results expressed as mean ± SEM, * *p* < 0.05, ** *p* < 0.005, *** *p* < 0.0005. C: -chow, HFD-V: HFD-vehicle, HFD-L: HFD with liraglutide, HFD-C: HFD with pre-conception switch to chow, HFD-PC: HFD switched to chow once pregnancy was confirmed.

The results of the high-resolution ^1^H NMR spectroscopy were categorized into metabolomic profiles of placentas, according to broad categories of metabolites based on redox and energetics (NADH, NAD, AMP, ATP, phosphocreatine, creatine), carbohydrate metabolism (lactate, glucose, glycogen) Tricarboxylic acid (TCA) Cycle (fumarate, succinate), lipid metabolism metabolites (choline, phosphocholine, carnitine, acetate, acetyl carnitine), and amino acid metabolism (glutamate, glutamine, aspartate, alanine) (Figure 3).

In terms of redox and energetics metabolic constituents, there was increased NADH abundance in the HFD-V group compared to the control group (*p* < 0.05) (Figure 3(Ai)). Liraglutide as well as all of the dietary modifications (HFD-L, HFD-C, HFD-PC) led to normalization of NADH, similar to the control, although not statistically significant. There was no difference in the concentration of the total adenine nucleotide pool constituents (AMP, ATP, Figure 3(Aiii,Aiv)) or energy reserve biomarkers (phosphocreatine, creatine, Figure 3(Av,Avi)) between groups. TCA cycle constituent concentrations (fumarate and succinate) were comparable between the groups (Figure 3(Biv,Bv)).

Glucose and glycogen concentrations were comparable between all groups, and there was no difference in lactate concentration between the HFD-V and control groups (*p* > 0.05). However, pre-pregnancy liraglutide intervention significantly increased placental lactate concentration compared to the control or HFD-V groups (*p* < 0.05, *p* < 0.01, respectively, Figure 3(Bi–Biii)).

There were no differences in choline or phosphocholine expression between groups (Figure 3(Ci,Cii)). Decreased expression of carnitine was detected in the HFD-V and HFD-PC groups compared to the control (both *p* < 0.05, Figure 3(Ciii)). Acetate expression was similar between the HFD-V and control groups, but both the HFD-L and the HFD-C groups had elevated acetate levels compared to HFD-V (*p* < 0.05 and *p* < 0.005, respectively, Figure 3(Civ)). Acetyl carnitine abundance was significantly lower in the HFD-PC group compared to the HFD-L group (*p* < 0.05, Figure 3(Cv)) but was similar between the C, HFD-V, and HFD-C groups.

The most significant metabolic impact of HFD and liraglutide intervention was observed in amino acid metabolism intermediates. Glutamate levels were lower in the HFD-V group compared to both the control and HFD-C groups (both *p* < 0.05, Figure 3(Di)). Glutamine levels were significantly lower in the HFD-V group compared to the control, HFD-L, or HFD-C groups (*p* < 0.001, *p* < 0.05, *p* < 0.005, respectively, Figure 3(Dii)). The HFD-PC group also had significantly lower glutamine levels than the control or HFD-C groups (*p* < 0.005, *p* < 0.05, respectively). Aspartate levels in the HFD-V group were significantly lower than the HFD-C group (*p* < 0.05, Figure 3(Diii)). Alanine levels were increased in the HFD-L group compared to both the control (*p* < 0.05) or HFD-V groups (*p* < 0.005, Figure 3(Div)).

## 4. Discussion

To our knowledge, this study is the first to compare the impact of weight loss in the pre-conception period, with the GLP-1 receptor agonist, liraglutide, or diet change, on placental inflammation, oxidative stress, and metabolome in a rodent model. This study found that maternal obesity caused significant changes in placental physiological profile, including oxidative stress (increased *NOX4*), inflammation (increased *IL-6*), and dysregulated metabolism. Intervention with liraglutide before conception in obese dams led to normalization of gene expression of some metabolic regulators (*PGC1alph*a) and metabolic intermediates (glutamine, alanine, lactate, acetate); however, it did not eliminate oxidative stress nor inflammation, which could be explained by the significant gestational weight gain observed in the group exposed to liraglutide. Pre-conception dietary change exerted similar effects to liraglutide treatment in restoring placental metabolic profiles. In addition, this intervention also reduced HFD-induced placental oxidative stress and inflammation. In contrast, diet modulation during pregnancy in obese dams showed signals of stress, despite observing no change in placental size in the HFD-PC group. These mice had worse glucose tolerance, as well as signs of placental stress, including low glutamine, acetyl carnitine, and carnitine levels, which have been previously shown to be associated with growth restriction and reduced fatty acid metabolism [25,26,27]. Further, the reduced number of surviving offspring after birth in the HFD-PC group further supports a greater degree of placental dysfunction. These findings suggest that a sudden diet change with relative caloric restriction in pregnancy may act as an acute “stressor” with detrimental outcomes.

The positive benefit of liraglutide therapy on placental metabolism occurred despite increased gestational weight gain. In the absence of diet change during pregnancy, mice treated with liraglutide had significant gestational weight gain compared to mice maintained on an HFD throughout, such that late gestational weight and concomitant glucose intolerance were similar between the HFD-fed groups, regardless of pre-conception liraglutide administration. This “catch-up weight gain” has been described by our group previously [11] and demonstrates that pre-conception weight loss is beneficial with regards to fertility and glucose tolerance, but following drug cessation, without concomitant diet modulation, rapid rebound weight re-gain occurs. This was not observed in the groups with diet change in either pregnancy or in the pre-conception period. It is well known that cessation of GLP-1 receptor agonists, in the absence of controlled caloric intake, induces weight regain secondary to increased hunger signals and palatability for higher-caloric foods [28]. Gestational weight gain is a known risk factor for complications in human pregnancy, such as gestational diabetes [29] and pre-eclampsia [30]. It can be inferred that this weight regain underpins the findings of increased inflammatory markers, with placental inflammation an early sign of dysfunction. Metabolic changes are often a response to inflammation and therefore maybe a secondary manifestation and thus may explain the different metabolic profiles in the placenta of HFD-L mice compared to HFD-V mice. The alterations in placental health with maternal weight reduction, induced with or without liraglutide use pre-pregnancy, suggest the critical role of the maternal endocrine and metabolic milieu at the outset of pregnancy but that gestational weight gain and its associated dysfunctional metabolic changes can significantly impact the later stages of pregnancy and therefore pregnancy outcomes.

Our study demonstrated placental dysfunction due to maternal obesity, marked by upregulated *FAS*, *IL-6*, and *NOX-4*, increased NADH, and reduced levels of amino acids (glutamate and glutamine, aspartate) and lipid metabolites (carnitine). Broadly, changes in placental structure and function due to maternal obesity have been previously described, though not with as much detail on the metabolome and different types of interventions as our study [31]. While TCA-cycle metabolites remained unchanged, secondary recruitment of amino acids as energy sources is indicative of metabolic stress. Reduced essential amino acid levels are associated with impaired fetal growth [32,33]. While considered ‘non-essential’, glutamate and glutamine become conditionally essential during pregnancy, with lower levels associated with smaller litter sizes and growth restriction [34,35]. Our findings suggest that obesity negatively alters the availability of these amino acids. Additionally, depleted fatty acid markers, such as carnitine and acetate, suggest increased lipid utilization in response to higher energy demands in the obese group, consistent with the existing literature [26], and increased *FAS* expression. Acetyl-CoA is used to produce ketone bodies as an alternative fuel source when required [36], and the placenta allows free passage of ketone bodies as a fetal fuel source [37]. The greater level of energy requirements and therefore lipid utilization by HFD-V group may explain the lower levels of lipid metabolites.

The placenta in the pre-pregnancy liraglutide group showed reduced *FAS* and *PGC1α* expression, indicating a lower need for lipid synthesis and storage, consistent with the effects of GLP-1 receptor agonists on lipid pathways observed in other studies [17,38,39]. The higher levels of amino acid and carbohydrate metabolites support this shift in metabolism. PGC1-α is involved in regulation of cellular energy metabolism, stimulating mitochondrial biogenesis and regulating carbohydrate and lipid metabolism [40]. This finding is consistent with the known effects of maternal diabetes on offspring PGC1-α gene expression [41]. Conversely, GLP-1 receptor agonist therapy was shown to upregulate PGC1-α expression [40]. In our study, placental tissue was not under direct exposure to liraglutide (treatment was ceased prior to conception), and maternal body weight and glucose levels had caught up to those of the obese group by late gestation, therefore explaining why *PGC1-α* expression was not elevated but rather reduced in late gestation. Oxidative and inflammatory markers, *NOX-4* and *IL-6*, were similar in the HFD-L and HFD-V groups, consistent with the known obesogenic effects of an HFD in pregnancy. Elevated levels of *IL-6* are associated with increased miscarriage and preterm birth, likely playing a modulating role during embryo implantation and placental development [42,43]. *NOX4* is also involved in placental redox reactions, with upregulated levels observed in preeclampsia [27,44]. Placental downregulation of *NOX4* and *IL6* seen in the pre-pregnancy diet switch group suggests a favourably altered inflammatory environment compared to the placentas of obese mothers. In keeping with the above changes, placental size, which is inversely associated with nutrient availability and metabolic stress [45], was greater in the HFD-C group compared to the HFD-L group. This may be due to the central effect of GLP-1 receptor agonist therapy, known to induce appetite suppression and therefore caloric restriction, which may account for placental size discrepancy when present during early placentogenesis [46]. Interpreted in this context, coupled with the overall improved weight and metabolic status of the HFD-C offspring, as well as our previous results showing improved fertility and fecundity [11], pre-pregnancy weight modulation with diet modification sustained throughout pregnancy is the most effective strategy to improve maternal and offspring health. The caveat to this is, of course, that in humans, maintaining consistent weight loss through diet alone is challenging, especially in the context of pregnancy [44].

There were several limitations to our study. First, liraglutide had to be stopped prior to pregnancy, given the known teratogenic properties previously described, and we did not include a group of mice with liraglutide together with diet modification to chow, as there was ethical concern that the total weight loss would be too severe, leading to physiological stress, negatively impacting well-being and fertility. Finally, there are inherent differences in placental structure between humans and mice, which should be considered in this study. Both human and mouse placenta are hemochorial although with different structures, and the trophoblast cell lineage develops through similar pathways, with an invasive branch facilitating implantation and an exchange branch establishing the syncytiotrophoblasts in both species [47]. However, the mouse placenta differs from the human placenta in some morphological characteristics, in that the human placenta has a more elaborate and sophisticated system for endocrine signalling and nutrient exchange [48]. Nonetheless, mouse models have the benefit over human studies of controlled experimental design, short gestational periods, and multiple gestations while limiting confounding effects of genetic and postnatal environmental influences [4]. Maternal serum, specifically circulating placental factors, was not examined in this study.

Despite these limitations, the clinical implications of these findings are apparent: placental health is impacted by an obesogenic milieu but can be modified by weight loss. Pre-pregnancy weight optimization by either diet or GLP-1 therapy does have benefits, with GLP-1 therapy effective even in the face of ongoing “poor” diet. However, upon drug cessation with ongoing HFD, this benefit is not sustained as pregnancies progress, and rebound weight re-gain occurs. The anti-inflammatory effect of GLP-1 therapy on the placenta is lost, while the beneficial metabolic changes may persist for longer. Diet change pre-conception seems to be the most effective intervention in terms of anti-inflammatory/oxidative stress impact on the placenta and its metabolic health. However, diet change in pregnancy, despite lower gestational weight gain, may constitute a stress for the pregnancy and may be deleterious.

## 5. Conclusions

Maternal obesity induces placental dysregulation of key metabolic signatures, ameliorated by weight loss prior to pregnancy. Liraglutide in the pre-conception period, despite being useful to improve fertility and maternal health, improved only metabolic aspects of placental health. The positive effects of liraglutide in early pregnancy seem to be negated by increased gestational weight gain and its metabolic and inflammatory sequelae by late gestation. It was thereby less effective over the course of pregnancy than diet modification before pregnancy when sustained throughout gestation. Although further studies are needed to confirm placental metabolite profiles after pre-pregnancy weight loss, our findings suggest that pre-conception weight modulation abrogates the deleterious effects of maternal obesity on placental health.

## Figures and Tables

**Table 1:** Mouse-specific primers used in quantitative real-time qPCR.

Gene	Forward Primer Sequence (5′-3′)	Reverse Primer Sequence (3′-5′)
18S	ACCGCAGCTAGGAATAATGGA	GCCTCAGTTCCGAAAACC
PGC1 α	CTCTCAGTAAGGGGCTGGTT	ATCCACTCTGACACACAC
FAS	TGCTCCCAGCTGCAGGC	GCCCGGTAGCTCTGGGTGA
PPARα	GGGCTCTCCCACATCCTT	TGGTCTTCAGGGCAATGTCG
NOX 4	AGTCTTAACCAGACATCATCC	CAGAAATCCAAATCCAGGTC
IL-6	AGACAAAGCCAGAGTCCTTCAG	GAGAGCATTGGAAATTGGGGTAGG

**Table 2:** Anthropometry, IPGTT, and fertility outcomes results in late gestation for HFD-PC group.

Anthropometry	C (Mean ± SEM)	HFD-V (Mean ± SEM)	HFD-L (Mean ± SEM)	HFD-C (Mean ± SEM)	HFD-PC (Mean ± SEM)	*p* Value
**Preconception body weight (g)**	23.4 ± 0.3	27.4 ± 1.1	24.8 ± 0.8	23.3 ± 0.5	25.1 ± 0.6	**<0.0001**
**Late gestation body weight (g)**	28.2 ± 0.9	33.2 ± 0.9	31.3 ± 0.9	27.7 ± 0.4	28.8 ± 0.6	**<0.0001**
**Gestational Weight Gain (g)**	5.7 ± 0.5	5.3 ± 0.7	6.8 ± 0.6	5.0 ± 0.4	3.2 ± 0.4g	**0.006**
**Late gestation IPGTT AUC (** **mmol/L/min** **)**	81.7 ± 4.5	122.3 ± 7.1	109.5 ± 11.3	83.8 ± 7.2	98.3 ± 7.0	**<0.0001**
**Serum insulin (mU/L)**	2.9 ± 0.6	1.9 ± 0.4	3.3 ± 0.5	3.0 ± 0.4	5.1 ± 0.5	**0.002**
**HOMA-IR**	0.9 ± 0.2	0.7 ± 0.1	1.5 ± 0.1	1.1 ± 0.1	1.6 ± 0.1	**0.005**
**NEFA (** **mmol/L)**	1.9 ± 0.1	2.5 ± 0.2	2.5 ± 0.2	2.0 ± 0.1	2.0 ± 0.1	**0.04**
**TAG (** **mmol/L)**	1.0 ± 0.1	0.6 ± 0.0	0.6 ± 0.1	0.8 ± 0.1	0.5 ± 0.0	**0.004**
**HDL (** **mmol/L)**	0.7 ± 0.0	0.5 ± 0.1	0.6 ± 0.1	0.6 ± 0.1	0.6 ± 0.0	0.6
**LDL (** **mmol/L)**	0.7 ± 0.1	0.7 ± 0.1	0.7 ± 0.1	0.9 ± 0.1	0.8 ± 0.0	0.4
**Number of foeti**	9.9 ± 0.7	7.3 ± 0.4	7.2 ± 0.4	8.3 ± 0.3	7.4 ± 0.4	**0.003**
**Foetal weight (g)**	0.3 ± 0.1	0.4 ± 0.1	0.5 ± 0.1	0.5 ± 0.1	0.4 ± 0.1	0.6
**Placental weight (g)**	0.11 ± 0.013	0.11 ± 0.021	0.11 ± 0.018	0.13 ± 0.021	0.11 ± 0.02	**0.01**
**Litter Size at birth**	6.6 ± 0.4	4.7 ± 0.4	5.6 ± 0.4	5.6 ± 0.4	5.2 ± 0.4	**0.03**
**Litter size at weaning**	5.8 ± 0.7	3.0 ± 0.5	3.9 ± 0.6	5.1 ± 0.5	3.7 ± 0.5	**0.008**

Results expressed as mean ± SEM, N = 8–10 per group. *p* value for ANOVA, group. Statistically significant *p* values in bold. IPGTT AUC, intraperitoneal glucose tolerance test area under the curve, HOMA-IR, Homeostatic model assessment for insulin resistance, NEFA, non-esterified fatty acid. TAG, triacylglyceride level. HDL, high-density lipoprotein. LDL, low-density lipoprotein. C, control. HFD-V, high-fat-diet-fed mice given vehicle. HFD-L, HFD-fed mice treated with liraglutide. HFD-C, HFD-fed mice changed to chow pre-conception. HFD-PC, HFD-fed mice changed to chow post-conception. Data reproduced with permission from [11].

## Data Availability

All data generated or analyzed during this study are included in this published article. Data are available on request to corresponding author.
